# AAPM medical physics practice guideline 13.a: HDR brachytherapy, part B

**DOI:** 10.1002/acm2.70118

**Published:** 2025-06-29

**Authors:** Susan L. Richardson, Arjit K. Baghwala, Gil'ad N. Cohen, Claire Dempsey, Bruce Libby, Christopher S. Melhus, Robin A. Miller, Daniel J. Scanderbeg, Samantha J. Simiele

**Affiliations:** ^1^ Swedish Medical Center Seattle Washington USA; ^2^ Houston Methodist Hospital Houston Texas USA; ^3^ NYU Grossman School of Medicine New York USA; ^4^ Calvary Mater Newcastle Hospital University of Newcastle Callaghan Australia; ^5^ University of Washington Seattle Washington USA; ^6^ Moffitt Cancer Center Tampa Florida USA; ^7^ Tufts Medical Center Tufts University School of Medicine Boston Massachusetts; ^8^ Northwest Medical Physics Center Seattle Washington USA; ^9^ UC San Diego San Diego California USA; ^10^ University of Alabama at Birmingham Birmingham Alabama USA

**Keywords:** brachytherapy, HDR, MPPG, practice guideline

## Abstract

The goal of this report is to assist the clinical medical physicist in assuring that key quality standards and practice considerations are met to ensure safe, reliable, and reproducible high dose rate (HDR) brachytherapy (BT) treatment. This guideline has been developed to provide appropriate minimum standards for such services. The secondary goal is to provide recommendations to the regulatory community from the experts to guide the adoption of regulations in the future. This MPPG is limited to iridium‐192‐based HDR brachytherapy and will not discuss electronic, low‐dose rate, pulsed dose rate brachytherapy, or any alternative radionuclide.

**Scope:**

This MPPG 13a report is divided into two parts. Part A has been previously published^1^ and describes the infrastructure and program design in the creation of an afterloader‐based HDR brachytherapy program. This publication, Part B, describes the clinical treatment processes including site‐specific imaging, planning, and treatment delivery. MPPG 13a Part B starts with the arrival of the patient to the clinic and concludes with emergency procedures and error mitigation.

**Disclaimer:**

It is the responsibility of all healthcare staff to be familiar with state and federal guidelines that may take precedence over AAPM recommendations that are provided in this report. Each health care facility may have site‐specific or state‐mandated needs and requirements that may modify their usage of these recommendations.

## 1 ABOUT MPPGS

The American Association of Physicists in Medicine (AAPM) is a nonprofit professional society whose mission is “Advancing medicine through excellence in the science, education, and professional practice of medical physics.” The AAPM has roughly 10,000 members and is the principal organization of medical physicists in the United States.

The AAPM will periodically define new practice guidelines for medical physics practice to help advance the science of medical physics and to improve the quality of service to patients throughout the United States. Existing medical physics practice guidelines will be reviewed for the purpose of revision or renewal, as appropriate, on their fifth anniversary or sooner.

Each medical physics practice guideline represents a policy statement by the AAPM, has undergone a thorough consensus process in which it has been subjected to extensive review, and requires the approval of the Professional Council. The medical physics practice guidelines recognize that the safe and effective use of diagnostic and therapeutic radiology requires specific training, skills, and techniques, as described in each document. Reproduction or modification of the published practice guidelines and technical standards by those entities not providing these services is not authorized.

The following terms are used in the AAPM practice guidelines:

- Must and Must Not: Used to indicate that adherence to the recommendation is considered necessary to conform to this practice guideline. **While must is the term to be used in the guidelines, if an entity that adopts the guideline has shall as the preferred term, the AAPM considers that must and shall have the same meaning**.
- Should and Should Not: Used to indicate a prudent practice to which exceptions may occasionally be made in appropriate circumstances.

## 2 INTRODUCTION

As described in the scope, this document is the second in a series of MPPGs focusing on HDR brachytherapy. The first was MPPG 13a Part A, which focused on minimum standards for the development of infrastructure and program design in an afterloader‐based environment.^1^ This report focuses on minimum standards in the clinical treatment processes, starting with the patient arriving at the facility and concluding with the patient departing after successful treatment.

## 3 PRE‐TREATMENT OVERVIEW

### 3.1 Pre‐treatment procedure

Before the patient arrives at a treatment facility, the patient may have already been seen by members of the same healthcare facility and/or had imaging studies performed at an outside institution. The physicist should review any pertinent previous treatments or imaging studies, which may assist in guiding technical recommendations ahead of time. Additionally, this information could be beneficial during a physics patient consult, if performed.^2^ This step may also occur at inter‐disciplinary chart rounds.

### 3.2 Pre‐insertion imaging

When starting or adding brachytherapy (BT) to a patient's treatment regimen, it can be helpful to have supplementary imaging information, which may include outside scans. Acquisition of additional imaging datasets can be challenging from a patient perspective in terms of time (travel and scheduling) and cost (another imaging procedure that may or may not necessitate insurance authorization). Nevertheless, procurement and review of any beneficial imaging scans ahead of treatment can help to guide the procedure. For example, if the patient has had either an independent diagnostic computed tomography (CT) scan or a CT acquired for external beam treatment planning, that dataset may be used to assist with pre‐determining needle quantity, needle mapping, and depth. Magnetic resonance images (MRI) may be imported to assist treatment planning and contouring of target volumes. Positron‐emitted tomography (PET) may be used for staging as well as for treatment planning for cervical cancer.^3^ Other imaging may be used to help guide pre‐surgical interventions or aid in applicator placement or target delineation.

### 3.3 Applicator preparation

As described in MPPG13a Part A, maintaining and updating an applicator inventory and having proper quality assurance (QA) processes for applicators will contribute to equipment integrity and reduce errors in the treatment planning process. Applicator insertion or placement may occur in the operating room (OR), imaging suite, or treatment room, depending on the workflow for a particular clinic. General recommendations *prior to applicator insertion* and simulation include the following, performed by trained member(s) of the BT team:

○ Confirmation of the appropriate applicator by the physician
○ Verification of applicator/needle/catheter sterility or cleanliness
○ Visual inspection of applicator(s) for damage or missing parts
○ Availability and functionality of all ancillary equipment (e.g., ultrasound (US) unit)
○ Documentation of the patient setup and immobilization technique, if any
○ Any supplemental information that needs to be used in future time‐outs.

Some applicators may be inserted with image guidance. A list of potential imaging modalities and techniques that may be used to place applicators is provided in Table 1.

**TABLE 1:** A list of site‐specific imaging recommendations. Site‐specific imaging for applicator placement, pre‐planning, planning, and pre‐treatment verification by body site is given. The term “clinical” refers to visual inspection, preferably by the physician. “Treatment Verification” refers to any imaging happening right before the patient's treatment to confirm applicator stability.

Site		Applicator placement imaging modality	Guidance for insertion and imaging	Simulation and planning imaging modality	Treatment verification modality
GYN	Vaginal cuff (Intracavitary) Cervical/Uterine (Intracavitary)	Clinical US CT MRI Fluoroscopy	If using implanted markers, verify insertion depth Verify applicators if the patient is transported	None if template‐based planning CT MRI US CT MRI	None/clinical CT 2D Planar imaging/fluoroscopy CT 2D Planar imaging/fluoroscopy
	Cervical/Vagina/Female urethra (Interstitial/Hybrid)	US CT MRI Fluoroscopy	Verify applicators if patient is transported	US CT MRI	CT 2D Planar imaging/fluoroscopy
Breast	Intracavitary	Clinical US	Verify device rotation, integrity, and filling Consider breath hold during the scan	CT	Clinical US CT
	Interstitial	US 2D Planar imaging	Consider breath Hold during the scan	CT	Clinical
	Non‐Invasive	Mammography	Have patient comfortable for long sessions	Mammography	Mammography
Skin		Clinical US Dermoscopy Optical Coherence Microscopy	Consider the use of transparency template to outline the target	Clinical CT MRI	Clinical
Intraluminal		Fluoroscopy/ Planar imaging Bronchoscopy Endoscopy	Check applicator post scope removal	CT (with breath hold for lung and hepato‐biliary applications) Fluoroscopy/Planar Imaging	2D Planar imaging/fluoroscopy
Prostate		US MRI CT Fluoroscopy	US only workflow may have no treatment verification Verify applicators if the patient is transported	US CT MRI PET (in conjunction with one of the above imaging modalities)	CT 2D Planar imaging/fluoroscopy

**FIGURE 1:**
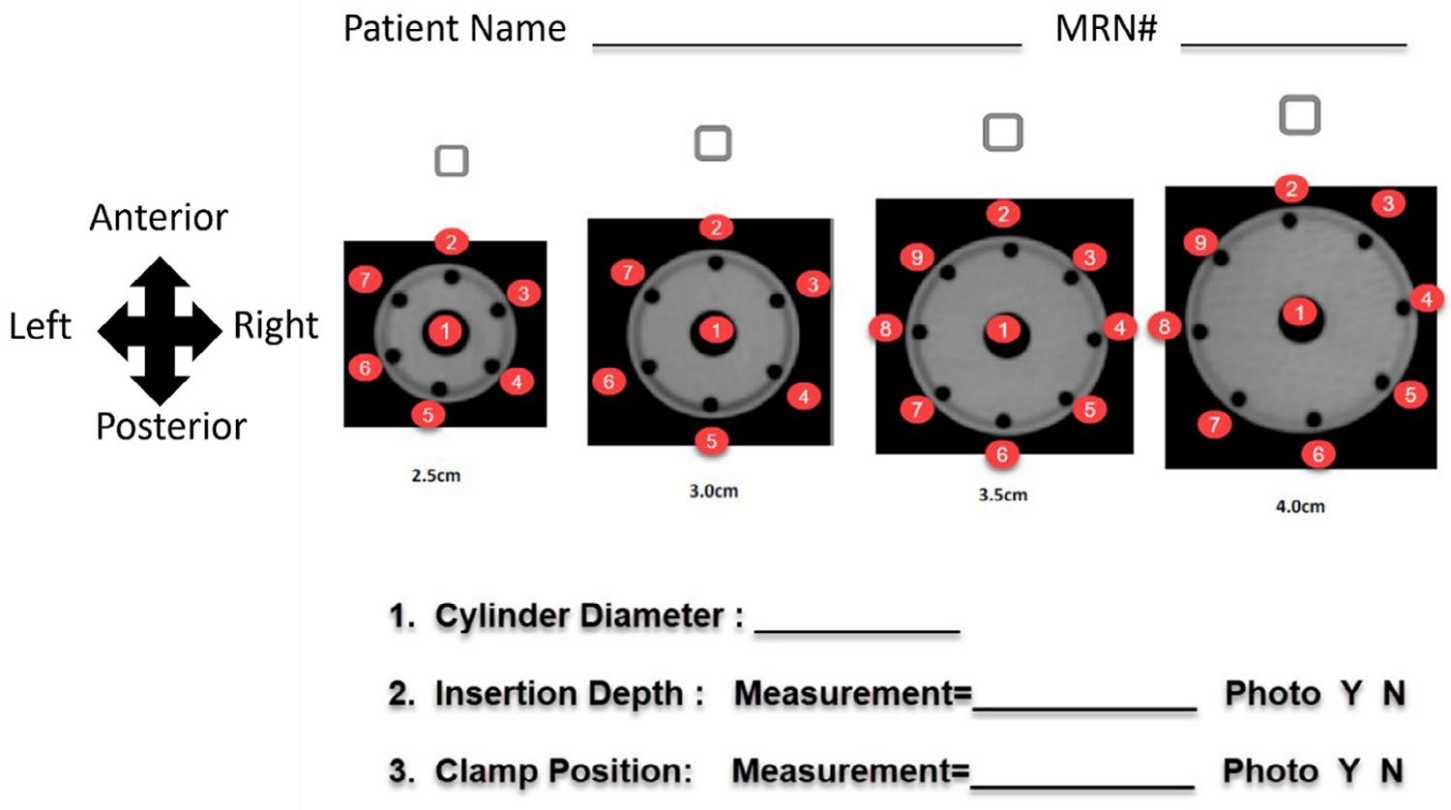
Example of a multi‐channel vaginal cylinder applicator documentation that is used both at the simulation and planning stages of treatment and becomes part of the patient's medical record.

After insertion, applicator geometry and/or orientation must be documented. Policies and procedures specific to the implant type must indicate the standard convention by which channels are numbered. For instance, a tandem and ovoid implant could designate: 1 (tandem), 2 (left ovoid), and 3 (right ovoid). For interstitial implants, directionality of the channel numbering technique (e.g., clockwise, from inside out) must be documented so that future planning and connection of the patient to the afterloader can be easily verified. An example of a multi‐channel cylinder naming convention is shown in Figure 1.

**FIGURE 2:**
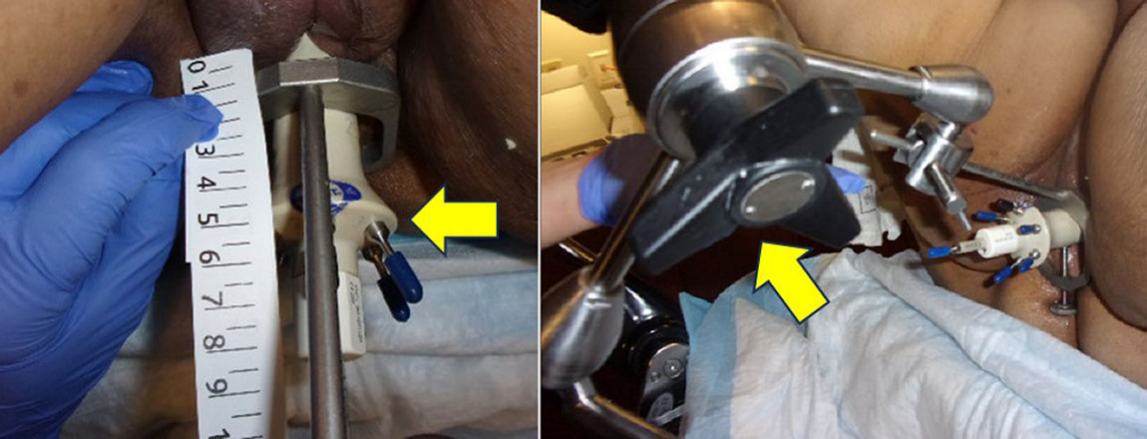
Example of photographic documentation of a multi‐channel cylinder (left arrow) including insertion depth and immobilization method (right).

Applicators may have external markings to orient the applicator. The setup documentation and photographs should be used to show patient and applicator orientation or distance from the needle end to template. Identification marks on the patient position and/or immobilization devices should be employed to document applicator placement and fixation. This is a critical step in the documentation process, as the individuals at the simulation may not be the same as those in the treatment room. Effective communication and careful documentation can reduce errors. Figure 2 shows a photograph of the insertion depth and immobilization techniques used for a multi‐channel cylinder.

Any remaining critical attributes related to initiating treatment planning, such as bladder fill volume or breast balloon volume, should be documented and verified by two individuals. Any other pertinent information related to the applicators or accessories should be logged and/or photographed in the patient's chart. Some facilities may wish to measure and document applicator/needle/catheter length at the time of simulation. In this case, the physicist or another trained individual can perform the measurements needed for treatment planning.

### 3.4 Immobilization

In contrast to external beam radiotherapy (EBRT), in which many types of patient immobilization devices are commercially available, BT has historically not immobilized patients to the same extent. However, geometric stability is critical when patients are moved from procedure rooms to imaging suites and then to the treatment vault, where the applicator can shift each time the patient is moved. Immobilization devices should be used when reasonable and appropriate. They can be as simple as mesh underwear that can hold gynecological (GYN) applicators in position to more complex systems, such as commercially available patient transportation systems in which a pneumatic device hovers over the couch top, or a couch top that detaches from the imaging table so that the patient and applicator are moved together.

### 3.5 Simulation

Patient imaging is usually required for planning and treatment verification purposes for HDR BT. A list of site‐specific recommendations for imaging techniques is provided in Table 1. The most commonly used imaging modality is CT. Other modalities such as US, MRI, orthogonal x‐rays, fluoroscopy, mammography, or cone beam computed tomography (CBCT) can also be used for patient and applicator imaging and planning. In general, CT slice thickness should be small enough for accurate applicator/channel tip identification but not so small that imaging artifacts occur—ideally reconstructed at 1–2 mm. Other recommendations state that CT for cervical cancer contouring should be less than 3 mm slice thickness, and the MR slice thickness should be between 3 and 5 mm.^4^ For treatment sites where respiratory motion may be an issue (breast, endobronchial, etc.) the use of breath hold simulation techniques may improve planning CT quality. Interstitial implants should be scanned obliquely or normal to the catheter plane when possible to decrease imaging artifacts. If the patient is moved between insertion and treatment, the recommendation to “verify applicators if patient is transported” is recommended in Table 1 to indicate a high risk of applicator movement and potential error.

### 3.6 Patient management

Certain aspects of managing BT treatments are beyond the purview of physicists, but physics personnel are often consulted. One example is that a lack of proper pain control can lead to patients moving more than advisable, and the applicator can then dislodge. Physics personnel should be consulted during the establishment of policies and procedures regarding BT patient care. The physicist can consult with the patient to explain their role in the treatment, what the patient can expect during treatment, and answer questions about radiation safety or other concerns that the patient may have.^2^ Physics personnel may be available to discuss patient care issues with staff, such as floor nurses or the anesthesia team, who may not be familiar with the procedures being performed. For patients that stay overnight with applicators in situ, it is imperative that the patient's care staff understand the nature of the implant and directives are communicated properly (e.g., interstitial gynecological (GYN) patients should not sit up in bed, catheters and needles need to be kept clean, etc.).

## 4 TREATMENT PREPARATION

### 4.1 Written directive and prescribing methods

A written directive, in the context of BT, is a documented order by an authorized user (AU) for the administration of radiation to a patient. NRC 10 CFR 35.40 has a list of the legal requirements of information to be present in a written directive.^5^ In the case of HDR brachytherapy, this includes:

1. Patient's Name
2. Radionuclide
3. Treatment target
4. Prescribed dose (dose per fraction, total number of fractions, total dose)

The written directive must be signed by the AU (electronically or otherwise) prior to initiation of treatment. Other information may also be included in a “prescription” that may be useful for verification during time out, planning and plan check. This prescription may include the basic tenets of the written directive and comments, notes, or guidelines. Optional items can include applicator size, treatment length, laterality, dose constraints, bladder (and/or rectal) filling instructions, or other valuable information for planning purposes.

It is recommended that the written directive be electronic and in concordance with federal and/or state regulations.^6^ Table 4 in MPPG 11a provides recommendations for additional items to be included in a BT prescription.^7^

### 4.2 Treatment planning and image fusion

Treatment planning images can be imported into the planning system at this time. Image registration was briefly discussed in MPPG13a Part A.^1^ Fusion of ancillary images for treatment planning purposes can be aligned via bony anatomy, soft tissue, or implanted markers, depending on what is most appropriate for the site. Image fusion for applicator confirmation (such as after an overnight stay) is typically done on the applicator. If performed, the fusion or registration must be checked in the region of the applicator and treatment volume by a different (second) trained individual prior to treatment.

### 4.3 Applicator reconstruction

Reconstructing applicators, needles, and catheters in the planning system can be one of the most technically challenging aspects of the treatment planning process. This step involves identifying the anticipated pathway and direction for the source to travel within the treatment channel. Depending on the number of treatment channels and the imaging technique used, accurate identification and delineation can be difficult and lead to dosimetric mistakes and medical events if done incorrectly. An excellent guide for reconstruction in GYN BT is given by Hellebust et al., and many of these instructions can be applied to other treatment sites.^8^ For CT‐based planning, plastic or metallic needles may be easy to auto‐detect through built‐in tools in the planning system. The channel identification diagram (obtained from simulation) should be referred to during this process to avoid orientation errors. The dead space at the applicator tip, such as the observed length of a beveled needle edge to the tip of the first dwell position (which is imaging protocol dependent) and required offsets for the applicators, should have been determined during commissioning. Applicator dimensions and offsets should be reviewed as required by the vendor, noting that some applicator definitions can be source dependent, meaning, they should be rechecked after each source exchange. One example is the tandem and ring applicator described in Tanderup et al.^9^ Depending on window/level settings in the planning system, volumetric averaging can be a source of error when determining the first dwell position. A systematic method of applicator reconstruction based on commissioning processes must be established.

**FIGURE 3:**
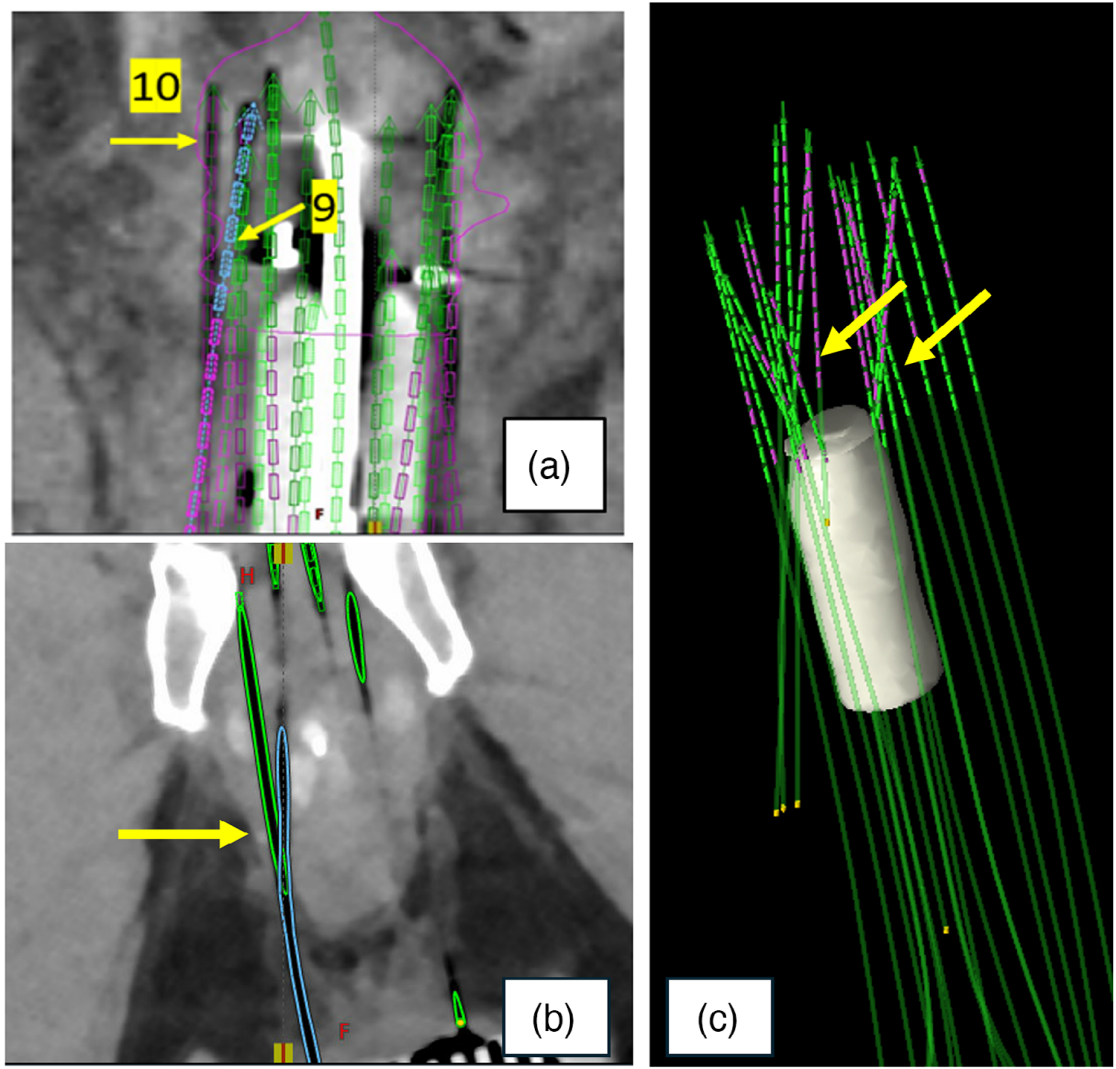
Examples of crossed needles with various applicators. Inset (a) shows both needles labeled 10 and 9. (b) shows needles crossing far from the tips. (c) is an example of multiple needles crossing in various planes.

In interstitial BT, plastic needles/catheters tend to deflect more than metallic ones if inserted without a stylet. Care should be taken in delineation of crossed needles, which may result in false trajectory detection by the planning system's automated processes. This may occur with manual digitization, as well. A 3D reconstruction of the needles should be reviewed for logical 3D spatial accuracy. In the 3D window, the needles can be rotated to an advantageous viewing plane for review of contiguity and viability. For example, complicated implants with crossed needles are shown in Figure 3. This necessitates the planning imaging to extend through the end of the template. Another technique to verify digitization of needles is to add the lengths of the visible needle outside of the patient to the measured depth inside the patient.

For GYN applicators with fixed geometry or fixed geometry components, utilizing the built‐in applicator library (discussed later) will reduce uncertainty and improve efficiency in the planning process. These may also be useful in datasets where there are significant metal artifacts. MRI typically leaves large voids of information where the applicators lie in situ, therefore comprehensive understanding of the applicator geometry prior to insertion in the patient is imperative to reduce errors.^10^ Applicator blooming in MRI can cause treatment channels to seem larger than they are. It is essential that the appropriate MR sequences (such as proton density weighted) are used for optimal visualization of the applicators.^8^, ^11^

Marker wires can be helpful in determining the orientation of the treatment channel arrangement (e.g., marker wire in Catheter #1) as well as the determination of the location of the first dwell position. Care should be taken to ensure the marker wires are inserted fully into the applicator/needle/catheter. Whatever method of catheter reconstruction is used, it must result in the positional accuracy of the source as described in MPPG13a Part A (2–3 mm depending on applicator and modality used). The planner must have an independent trained individual check the reconstruction prior to treatment, preferably before planning starts.

### 4.4 Target volumes and organs at risk

An important aspect of the treatment planning process, besides delineating tumor volumes and the organs‐at‐risk (OAR), is the naming of each structure. Readers should refer to AAPM TG‐263 and the updated recommendations to be published in TG‐263U1, which will include BT nomenclature.^12^ The philosophy for employing standardized nomenclature is to increase consistency between clinics and to improve contiguity in patient care. This MPPG recommends following standardized nomenclature recommendations such as TG‐263 and subsequent updates. Typical target volumes and OARs for each site are described in the site‐specific planning section in their respective tables.

### 4.5 Library applicators and templated plans

The use of library applicators and templated plans can improve standardization and efficiency in patient treatment planning. A library applicator is a model of the chosen applicator (or applicator components) that has been provided by the vendor to match the physical specifications and dimensions manufactured product. The use of library applicators provided by the vendor can reduce digitization and contouring uncertainties and standardize the critical position of the location of the first dwell point of the source. It can also improve consistency in defining secondary structures and reference points where scan parameters and window/level settings may influence delineation. It can reduce errors due to wrong applicator geometry, size, or rotation by leveraging direct visualization in overlapping the library contour with the imaged applicator. Applicator libraries must be acceptance tested and commissioned (as described in MPP13a Part A) to ensure accuracy.^13^ A clinical template plan or protocol is a standardized plan created by the end user that can include fixed structure names, points, clinical goals, and even dwell times. The use of templated or standardized plans can provide a uniform baseline for cases that change minimally (or not at all) from patient to patient. The use of standardized plans can reduce error introduction by miscalculation, mis‐delineation, or other mistakes. Templated plans can reduce the overall procedure time for certain treatments.^14^ However, despite the benefits offered by standardization of planning, a 2019 ABS survey reported that only 8% of practitioners used templated plans for postoperative endometrial BT in 2019.^15^

**FIGURE 4:**
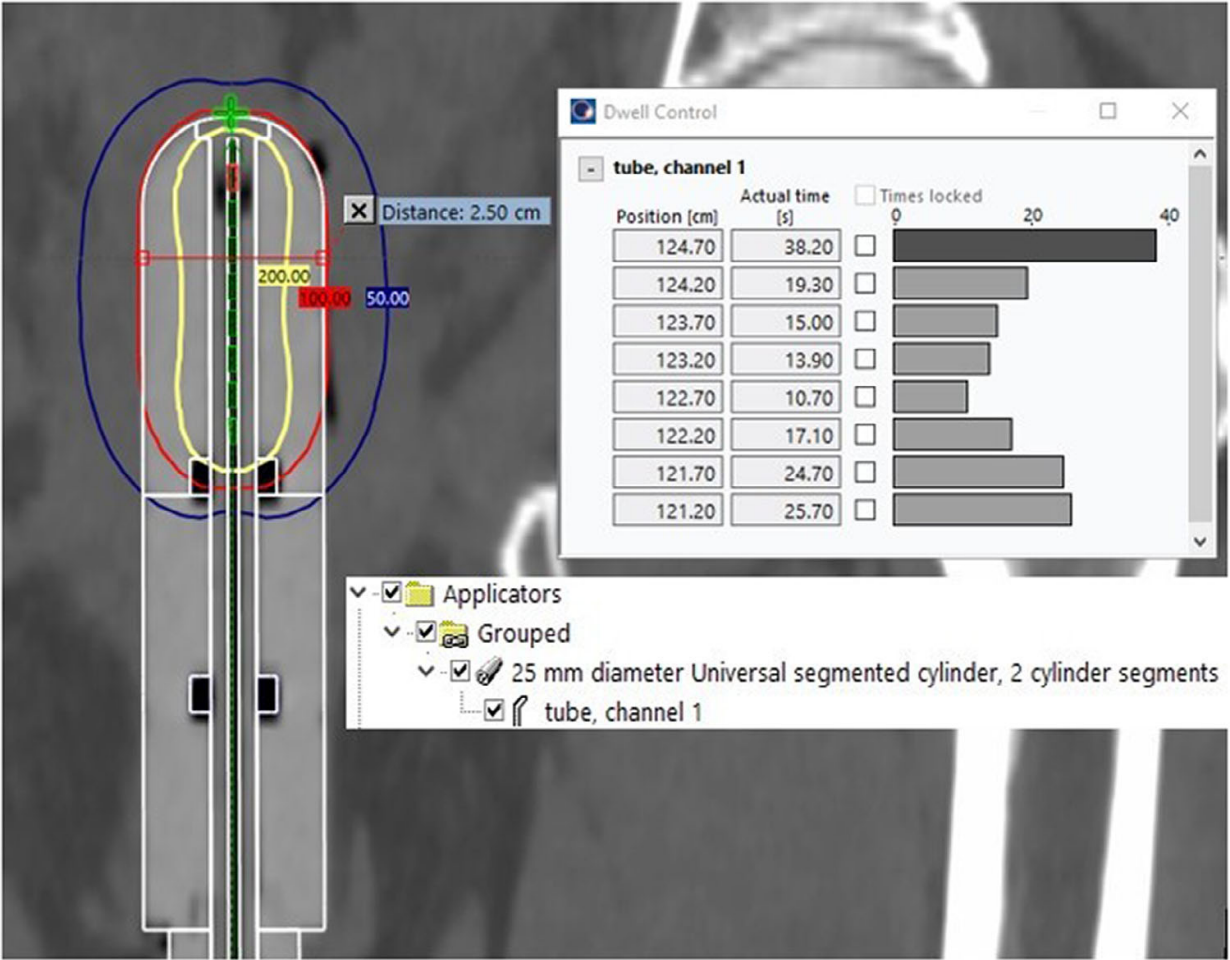
A solid applicator used in a templated plan. The applicator, the 25 mm diameter Universal Segmented Cylinder, is inserted into the plan and overlaid on the treatment cylinder (white rind), and the source pathway is already delineated. Minor adjustments can be made at this point to the dose distribution on a patient‐specific basis.

**FIGURE 5:**
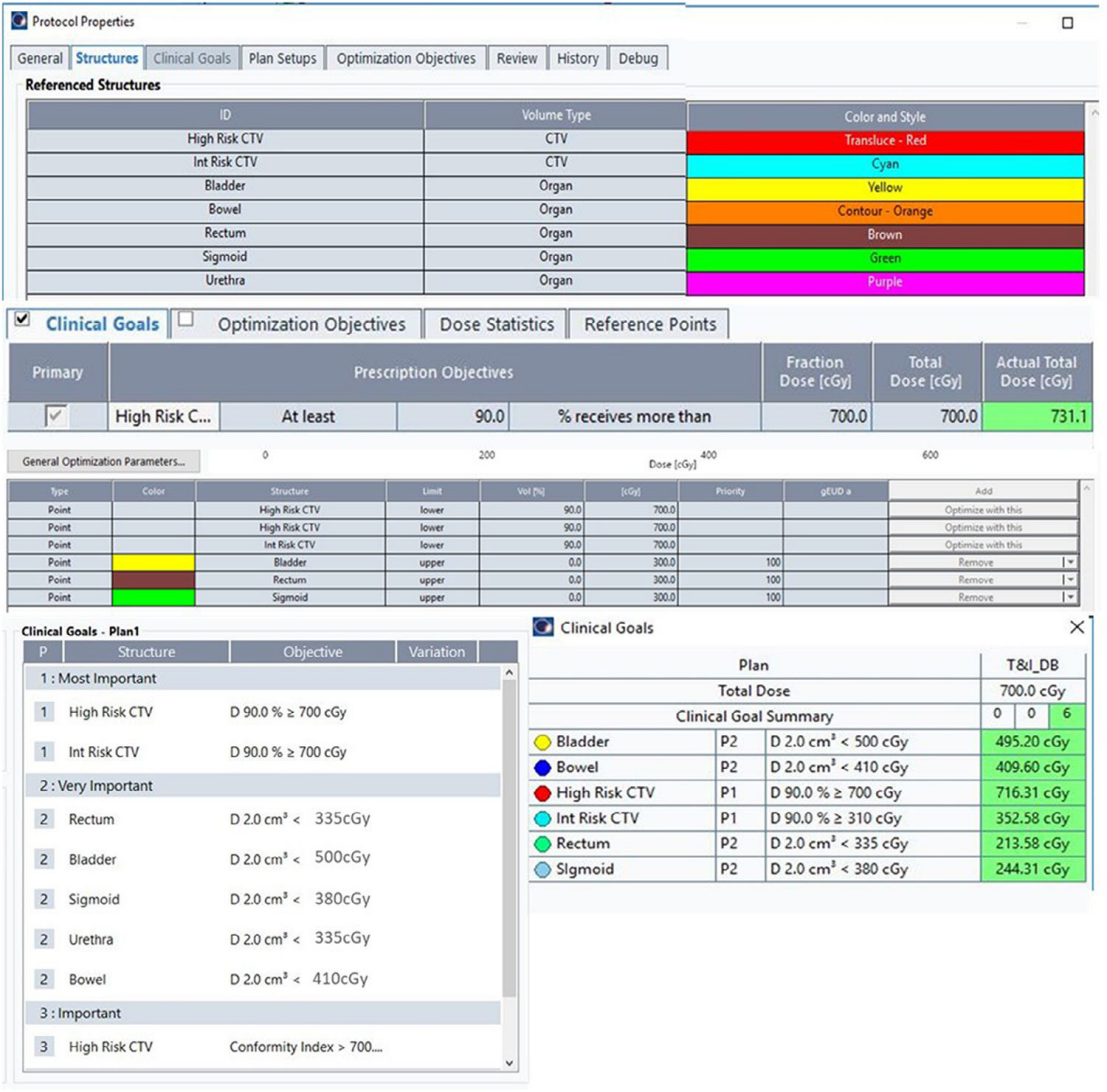
A clinical protocol for cervical HDR BT. Structure names, types, and colors can be templated (top), followed by clinical goals for the targets and structures (middle). Objectives for optimization for CTV/PTV structures are shown (bottom left) as well as dose constraints for OARs. Calculated dose metrics for a clinical plan are shown (bottom right).

It is recommended that templates and standardized plans be used whenever possible and practical, and it is strongly encouraged in curved applicator geometries. A screenshot of library applicator use is shown in Figure 4, and a templated plan in Figure 5.

### 4.6 Complicated scenarios

An extraordinarily complex treatment plan may contain more treatment channels than are physically capable by the afterloader in a single delivery. Most vendors allow a plan to be “split in two” and be composed of two separate deliveries within the system. This occurs most commonly in the treatment of skin, and may also occur in large interstitial GYN or sarcoma cases. The vendor‐recommended process should be tested ahead of time, and the documentation confirmed. Standardizing channel numbering and checking labeling and connections with clearly labeled diagrams is even more critical due to the potential of treatment overlap and increased risk of mistakes.

Multiple sites treated simultaneously can add a new level of rigor and complexity to an already difficult procedure, for instance, concurrent bilateral breast treatments. Extra care should be taken to ensure laterality and the proper treatment side. It may be useful to use different‐sized applicators or a different number of treatment channels between sides to differentiate treatment sites, if at all possible. If treating the skin surface, multiple lesions or lesions adjacent to previously treated ones can lead to mistaken identity and mistreatment. Thoroughly annotated pictorial documentation of the treated sites can go a long way in preventing such errors.

### 4.7 Radiobiology

Due to the targeted nature of BT dose distributions and the invasive treatment technique, clinical treatment guidelines often recommend hypo‐fractionated prescription regimens for HDR BT. This can be either as a stand‐alone treatment, as salvage therapy, or as part of a treatment protocol involving a combination of EBRT and BT. As such, radiobiological considerations can be incorporated in overall dose assessment to both the tumor and nearby OARs in many cases. Radiobiological calculations and models must be used for curative cervical BT treatments, specifically when combined with an EBRT and BT protocol. In order to determine expected clinical outcomes and evaluate the potential for radiation‐based treatment complications for key volumes (both target and OAR) and dose points, the total radiation dose from both the EBRT and BT doses must be converted to 2 Gy dose equivalent (EQD2) in order to be accurately combined.^16^ Other GYN cases where radiobiological calculations should be performed are for interstitial and vaginal primary. Radiobiological calculations for other sites, such as prostate, can be performed but are not mandatory. Commercially available TPSs do not provide biologically based dose reporting or EQD2 reports for BT. Therefore, most users rely on calculation spreadsheets (such as those available at https://www.americanbrachytherapy.org) and home‐grown scripts for these calculations and should ensure they are properly commissioned and locked prior to clinical use.

## 5 SITE SPECIFIC PLANNING GUIDELINES

The following site‐specific information contains tables to summarize key information, metrics, imaging recommendations, resources, and concerns based on each anatomic site described. While the discussion is not exhaustive, it presents baseline information and minimum concepts to be considered. Tables 2, 3, 4, 5, 6 are similar to each other but not identical based on some of the different complexities surrounding various treatment sites. Common treatment prescriptions and fractionation patterns are provided as a guide and are not all‐inclusive. For example, interstitial GYN fractionation and dose patterns may depend on the EBRT doses received by the patient and the subsequent EQD2 calculations. Implant geometry may be more favorable in one fraction than another, and hence, doses may change across treatment fractions.

**TABLE 2:** Gynecological treatment table. Each column describes a different applicator type. Typical applicators, target volumes and OARs, treatment regimens, and special considerations are provided. Fx stands for fraction.

	Intracavitary	Interstitial	Hybrid
# of Channels	1–9	1–99	1–99
Applicators	Cylinder, T&O, T&R, Y‐applicator, multi‐ channel cylinder	free hand or templates	Cylinder, T&R, tandem with fenestrated ovoids, needle templates combined with cylinders and/or tandems
Possible target volumes or prescription points	GTV, CTV_IR, CTV_HR, Points A and B, Cylinder: distance from applicator (5 mm), applicator surface		
OARs	bladder, rectum, vaginal vault, sigmoid colon, small bowel, urethra		
Common treatment regimens	Vaginal Cuff: 600–700 cGy x 3 fx 400–500 cGy in 4–5 fx At the surface or 5 mm	Vaginal: 600–700 cGy x 3	Cervix: 700 cGy x 4 fx 800 cGy x 3 fx
	Cervix: 700 cGy x 4 fx 800 cGy x 3 fx	Cervix: 500–600 cGy in 4–5 fx	
Special considerations	Left and Right ovoids are labeled and connected properly	Simulation pictures and diagrams Proper catheter labeling and connection to the afterloader Securing of needles	
	Patient management if transported between fractions—medications, applicator verification, intrafraction motion bladder and rectal prep and protocols		
References	ABS guidelines,^17^, ^18^ GEC ESTRO guidelines,^16^ AAPM TG‐303,^19^ EMBRACE ^20^		

**TABLE 3:** Breast treatment table. Each column describes a different applicator type. Typical applicators, target volumes and OARs, treatment regimens, and special considerations are provided.

	Interstitial	Balloon applicator	Strut applicator	Non‐invasive
# channels	1–99	1–3	1–11	2
Possible target volumes or prescription points	CTV, PTV_EVAL, applicator surface			
OARs	skin, chest wall, ribs, lung, heart			
Common Treatment Regimens	340cGy x 10 fx 750cGy x 3 fx 400cGy x 8 fx			340cGy x 10 fx
Special considerations	Edema/Seroma	Edema/Seroma Applicator rotation, Applicator integrity, skin bridge, distance to chest wall		Edema/Seroma Patient movement, Applicator integrity
References	NSABP 39 and RTOG 0431,^21^ TRIUMPH‐T,^22^ ABS guidelines^23^			

**TABLE 4:** Skin treatment table. Each column describes a different applicator type. Typical applicators, target volumes and OARs, treatment regimens, and special considerations are provided.

	Flap or mold‐based	Interstitial	Cone based
# Channels/ geometry	Parallel catheters with 1 cm spacing		Various diameters, from 1 to 3 cm
Applicators	Catheter flap‐based Custom or commercial	catheters or needles	Leipzig Valencia
Possible target volumes	GTV, CTV = GTV + 10 mm, CTV = PTV		GTV+radius
Prescription points	Prescribe up to 5 mm depth	Can prescribe beyond 5 mm	Prescribe to a 3–4 mm depth
Treatment regimens	300cGy x 18 400cGy x 12 500cGy x 8	250–400cGy x 14	700cGy x 6 500cGy x 10
Treatment localization	Clinical CT	CT	Clinical
Special considerations	Conformance with the surface Scatter effects Catheter verification	Swelling and button constriction	Cone placement and immobilization Flattening filter and cap presence
References	ABS Consensus statement^26^ GEC‐ESTRO Recommendations^27^		

**TABLE 5:** Intraluminal treatment table. Each column describes a different applicator type. Typical applicators, target volumes and OARs, treatment regimens, and special considerations are provided.

	Bronchus	Esophagus	Bile duct
	1 channel: linear geometry can be used as an approximation		
# Channels/ geometry	2 channel: V or Y configuration	Target may extend laterally past the GE junction	2‐3 channel: Variable geometry
Applicators	Single catheters (w or w/o radiopaque tip) Centering applicator (Balloon‐based for distal bronchi)	Centering applicator Bougie style Balloon based	Single catheters (w or w/o radiopaque tip)
Possible target volumes or prescription points	5 or 10 mm from the applicator(s). Coverage includes the tumor with 1–2 cm proximal/distal margins		
OARs	Heart	heart esophageal mucosa	duodenum
Treatment regimens	500‐1000 cGy x 1fx 1 ‐4 fractions total	500 cGy x 2fx 800–1500 cGy in 1–2fx	1500‐2000 cGy in 2–3 fx
	Multiple insertions w/1 tx/insertion		
Treatment localization	X‐ray / Fluoroscopy		
Special considerations	Centering applicators can be used if surface dose is a concern. Catheter identification in a multi‐catheter setup. Breath hold at simulation may be needed for catheter digitization. Anesthesia monitoring required	Centering applicators can be used if surface dose is a concern.	High curvature of the catheter may result in the treatment channel being compromised and unusable Catheter identification in a multi‐catheter setup. Breath Hold at simulation may be needed for catheter digitization.
References	ABS guidelines^32^	ABS guidelines^33^	Skowronek and Zwierzchowski^34^

**TABLE 6:** Prostate treatment table. Each column describes a different imaging modality for treatment planning. Typical applicators, target volumes and OARs, treatment regimens, and special considerations are provided.

Treatment planning imaging modality	US (± MR Informed)	CT (± MR Informed)	MR	MR Guided
# Channels	Typically 14–18 (prostate bed ∼ 6–10)			
Applicators	Plastic (use stylet), titanium, or stainless‐steel needles		MRI safe or MRI conditional needles and templates	
Implant Guidance Imaging	Needles placed using TRUS guidance US QA should be performed annually			MRI‐guided insertion
Possible target volumes or prescription points	PTV, dominant intraprostatic lesion (DIL)			
OARs	rectum, urethra, sigmoid, small bowel, bladder			
Common treatment regimens	Monotherapy: Definitive: 1350 cGy x 2 fx over 1–2 weeks or ≥ 4 h Salvage: 1100 cGy x 2 fx over 1–2 weeks or ≥ 4 h Boost: 1500 cGy x 1fx + EBRT; can incorporate HDR dose escalation to DILs.			
Special considerations	Patient in extended lithotomy position for the duration of the procedure Shielded procedure room required Anesthesia monitoring required	Patient/applicator immobilization for the duration of the treatment planning/delivery process Pre‐treatment verification should be performed if the patient is moved prior to treatment		
References	AAPM TG‐303^19^ RTOG 0924^36^ GEC‐ESTRO prostate BT guidelines^37^ ABS prostate guidelines^38^			

### 5.1 Gynecological

Gynecological cancer treatment is typically the backbone of any BT department. The treatments range from simple single channel vaginal cylinders to complex multi‐channel hybrid intracavitary and interstitial devices. Each applicator will come with instructions for use from the vendor that include sterilization and geometrical information. These instructions should be implemented and followed. Total doses and fraction numbers vary to achieve recommended EQD2 doses. Site‐specific recommendations for GYN are provided in Table 2.

### 5.2 Breast

A variety of commercially implanted devices are available for the treatment of the breast. These are typically single‐use devices with 1 to 11 treatment channels. Free‐handed or templated interstitial implants are still used in some centers. A non‐invasive methodology using collimating applicators is also included in the table below. Site specific recommendations for breast BT are provided in Table 3.

Single entry devices have the advantage of requiring only one incision in the patient versus multiple needle insertions with interstitial. However, it requires special considerations due to the complex nature of the device itself. Single‐entry devices can fail with either balloon rupture or strut collapse, necessitating pretreatment imaging before each fraction to ensure device integrity. Single‐entry devices can also rotate within the cavity, leading to an incorrect dose delivery. Monitoring of seroma levels and the distance between the applicators or balloon surface and any OARs is critical, as the dose distribution may be unacceptable with anatomical changes.

Interstitial applicators need to be visually inspected to check for movement. Any swelling can cause irritation and pain at the ends of the catheter if the buttons that secure the catheter in place are too tight.

Non‐invasive breast BT uses conical applicators placed against the breast to deliver a dose to the tumor bed. These are heavily shielded applicators, and the dose delivery is based on the applicator, so the applicators should be inspected prior to each fraction for any signs of damage (e.g., from being dropped). The patient should be monitored carefully during treatment for any signs of motion, as fatigue and body position changes could cause the target area to shift outside the treatment field.

Additionally, the treatment area should be monitored during the course of their treatment for signs of swelling or seroma drainage, as these can affect the plan parameters.

### 5.3 Intraoperative BT (IORT)

IORT involves the placement of an applicator directly on top of the surgically exposed target area. Since the entire procedure is done in sterile conditions, applicators and transfer guide tubes should undergo QA prior to each case. Validation of the sterilization process should be done as part of applicator commissioning to ensure the integrity of the applicator at the time of treatment. It is recommended that standard channel lengths be used, and that standardized planning and channel assignment nomenclature be developed to simplify the treatment planning/QA process in what is already a high‐pressure environment.^24^, ^25^

### 5.4 Skin

Skin or surface BT is primarily utilized to treat nonmelanoma skin cancer or keratinocyte carcinoma. Generally, radiation is delivered in a geometry external to the body, either using a flap in conformance with the skin or with a conical, shielded applicator; however, interstitial techniques may be considered for more depth‐extensive disease. Site‐specific recommendations are provided in Table 4.

For external catheter or flap‐based techniques, radiation is delivered using parallel catheters in conformance with the skin, spaced at a uniform distance from the surface, generally 5 mm. Flexible, commercial flap molds may be utilized, or a custom‐built applicator can be constructed, for example, using a bolus for skin spacing and/or a thermoplastic cast for rigidity and anatomic conformance. GEC‐ESTRO recommendations suggest varying the spacing from the skin as needed (up to 1 cm) to alter the gradient of the dose fall off, as long as there is good conformance between the surface and the mold.^28^ Treatment planning may be performed using CT, and treatment verification may be performed clinically or with volume‐based imaging. As this technique has the potential to require multiple catheters, catheter diagrams, photographs, and labeling are essential for patient safety and treatment reproducibility. While this approach is useful in conforming the applicator geometry to the treatment site, it can be a time‐consuming and complex treatment to deliver in the setting of large treatment areas and a resource‐limited clinic. Further, some studies suggest that HDR BT may be associated with higher OAR and integral dose than EBRT techniques in highly curved treatment geometries.^29^, ^30^

For conical applicators, extensive commissioning is required to validate the output of the applicators to ensure accuracy in treatment planning. A critical step involves characterizing the appropriate dwell position for the source to maximize applicator output from the shielded applicator, which may vary depending on a horizontal or vertical source position relative to the shielded cone. This typically requires radiosensitive film to assess dose homogeneity inside the applicator. Some applicators may be compatible with ion chamber‐based systems to validate output, while others may allow constancy tests and output factor verification using an adapter integrated with a well chamber. Once characterized, treatment planning is generally performed clinically using look‐up tables or 1D calculations, however, some institutions have used 3D printing with heterogeneity corrections.^31^ Applicators may integrate flattening filters or plastic caps to shield the patient from electrons generated in high‐Z material components. Processes should be in place to assess for the presence or absence of these components as part of treatment delivery.

For tumors that extend more than 5 mm from the surface, it may be necessary to use an interstitial treatment technique to treat deep‐seated targets. Treatment catheters can be visualized using CT guidance. As for the flaps described above, careful labeling and diagramming is necessary to ensure a reproducible connection to the afterloader.

### 5.5 Intraluminal

Intraluminal BT includes the placement of a small catheter into either the bronchus or the esophageal region of a patient. It can also include treatment of the bile duct in the liver. Intraluminal BT procedures usually require the assistance of an interventional radiologist or surgeon for catheter placement and present unique challenges. For endobronchial BT, the catheters are usually inserted into the nose through an endoscope, with either markers or stents used for verification of treatment location. Sometimes the catheter is marked physically on the exterior visible portion as a surrogate for target depth. Respiration and coughing can be an issue for simulation and treatment. If multiple catheters are used, each should be clearly identified and marked. Table 5 provides a summary of these considerations.

### 5.6 Prostate

Prostate HDR BT treatment is a team effort involving a multitude of different disciplines. The treatment can be performed in a shielded procedure room with remote anesthesia monitoring or in separate locations for applicator placement and treatment. When incorporating US imaging, routine QA should be performed per AAPM TG‐ 128. Localization and contouring methods can vary depending on treatment methodology. When incorporating dose escalation to dominant intraprostatic lesions, MRIs may be registered to either US or CT planning sets.^35^

### 5.7 Timelines

The timeline from the patient's arrival in the clinic to the end of their treatment will vary depending on the type of BT administered and the size of the team available. In general, the more applicator components and treatment channels used, the more complex and time‐consuming the procedure will be. As discussed in Prisciandaro et al., the addition of multiple imaging sets generally requires a longer time (45–90 min) to finalize the treatment plan.^39^ Having an image‐guided BT suite can reduce the timeline. Simultaneous contouring and applicator/needle/catheter reconstruction in a parallel workflow can improve efficiency.^40^ The team should consider the number of applicator sets necessary for an efficient workflow as well as the number of software licenses needed.

General timelines may be estimated as:

‐ 9 h from implant to treatment for interstitial GYN, reducing to 4 to 6 h for an image‐guided BT suite.^39^ Preplanning may also reduce timelines on subsequent fractions.^41^
‐6 to 7 h from implant to patient release for T&R GYN. One study found an average of 45 min for imaging, and 2.25 h for treatment planning.^42^
‐2.5 to 4 h for real‐time US‐guided prostate^43^
‐5 to 8 h for MRI‐based prostate^44^

Some patients may not be implanted and then simulated and treated on the same day, such as applicator‐based breast BT.

## 6 PRE‐TREATMENT QA AND TREATMENT DELIVERY

This section begins with the patient coming to the HDR treatment room for BT initiation. The patient is identified via two methods and brought into the vault or suite and a radiation survey performed. Daily quality assurance on the afterloader was described in MPPG 13a Part A and will not be discussed here.

### 6.1 Pre‐treatment image acquisition

Some patients are moved between fractions with the applicator in situ and obtain either in‐room imaging or a confirmation scan prior to treatment initiation. These verification datasets may be fused to the planning imaging set or reviewed off‐line to verify applicator position and/or integrity, and if the patient can be treated. Image registration was discussed in section 8.1.5 of MPPG13a Part A. Due to the high dose gradients in BT, small applicator or anatomical motion can lead to large changes in the target or critical structure doses. For example, in prostate BT, the prostate can move in relation to the needles, and the needles can also move in relation to the prostate and/or template, even with the use of fixation devices. As such, implant geometry should be verified prior to treatment.^19^ This can be accomplished by visual inspection/measurement, with the use of US, C‐arm x‐ray imaging, or more advanced imaging techniques. Care should be taken to verify both the internal alignment of the treatment channels with the target and the external alignment based on marks, measurements, and patient anatomy. A clinical judgment may be necessary to determine if it is appropriate to treat the patient if the applicator has shifted compared to planning. If ancillary imaging is required before treatment, two individuals should review the images and/or image fusion for acceptability. Discussions on uncertainties in applicator/needle/catheter positioning and subsequent doses can be found in Kirisits et al. and Hoskin et al.^45^, ^46^

### 6.2 Treatment Planning checks

As described in AAPM TG‐275, the development of an appropriate checklist for plan checking can be based on an FMEA of one's own departmental process, highlighting the steps assigned the highest risk.^47^ The TG‐275 report provides a comprehensive list of suggested items that can be checked. At a minimum, the following items must be checked in an initial HDR plan review by another independent trained individual (not the planner):

- Review of target volume (if any) and critical OARs within imaging data set (if any)
- Applicator/needle/catheter digitization, position of the first dwell position, treatment channel length and directionality, and treatment device correctness
- Channel mapping matches the simulation document (if any) or standard channel assignment
- Planned dose and/or DVH metrics and constraints, such as EQD2 matches approved written directive.

As will be described later, these items correlate with the highest risks of errors in BT. Other items that should be checked that are part of best practices are: dwell times are reasonable and smooth along a channel, unused or barely used channels are turned off to reduce transit dose, hot spots are minimal and occur in the target, optimization or inverse planning techniques are reasonable, and dose metrics are appropriate and match treatment site and accepted guidelines. AAPM MPPG 11a also has a similar section of recommended BT chart check items, though these were not risk‐management based.^48^ As described in MPPG 13a Part A, an independent dose calculation is optional.

### 6.3 Connection to afterloader and applicator check

Once in the treatment room, the applicators may be placed (if not previously), and the treatment channels connected to the afterloader via TGT. If the applicator/needle/catheter and TGT length were not measured at simulation, it may be performed at this time. For single or one‐time‐use devices that come sterile from the manufacturer, the combined applicator/catheter/needle and TGT length must be manually measured and verified for at least the first fraction. An exception to this is if the treatment unit measures and corrects for treatment channel length automatically. For reusable applicators, the combined applicator and TGT length should be checked annually as described in MPPG 13a Part A, and it is good practice to check once per patient. As will be described later, the total treatment length measurement is one of the biggest sources of error in HDR BT. Periodic spot checking of applicator/needle/catheter and TGT length is encouraged, as TGTs may stretch with time and use.

During the connection process, the treatment channels and any fixation devices should be inspected to ensure integrity and positioning (if the patient was transported with the applicator, needles, or catheters in situ). Photographs from the simulation can also assist in the verification of the applicator position. The connections should take place with the aid of the channel mapping diagram (if created) and documentation (such as Figure 1), which describes which TGTs should be used. TGTs should be visually inspected and have no kinks or obstructions. An independent trained individual (described in section 6.2 of MPPG 13a Part A) must double‐check the connection process.

### 6.4 Treatment console checks

After AU plan and Authorized Medical Physicist (AMP) treatment approval, the plan should be exported to the treatment console. At the treatment console, the imported plan should be checked for correct import and dwell time correction if the plan time is modified due to source decay at the console. The source strength should be checked if the plan is scaled.

### 6.5 Plan Documentation

The treatment plan documentation must be approved by the AU in the medical record and/or the record and verify system. This should include any ancillary calculations, such as EQD2 spreadsheets or secondary dose calculations, if performed. Typically, this will include isodose distributions, dose volume histograms, and technical delivery information. Photographs, catheter mapping diagrams, and checklists can also be recorded in the medical record.

### 6.6 Treatment and post‐treatment

After confirming all personnel have vacated the treatment room, a final time out must be performed and documented. Following the Joint Commission's standards,^49^ this should include a minimum of the patient's name, treatment procedure and site, applicator type and size, and any other patient‐specific critical information. The time‐out can be expanded beyond hospital standards and include fraction number and dose, bladder and rectal filling constraints, and so on. It is a good idea to include items to be verified that, if incorrect, would lead to a medical event. The time‐out should be standardized and include immediate team members. The time‐out is also a good time to check that individuals who are responsible for responding during an emergency procedure have their personal radiation dosimeters on.

The treatment must be initiated with the AU and AMP present, and the patient monitored during the entire treatment. The AMP and the AU (or trained physician designee) must be physically present at all times during the treatment.^50^ After successful completion of the treatment, the patient and afterloader must be surveyed per 10 CFR § 35.604.^51^ Documentation of the completed treatment delivery record should be placed in the patient's chart. Having a pre‐ and post‐treatment quality control checklist customized to the institution's workflow is recommended. Completion of these tasks should be documented after each step and recorded.

## 7 EMERGENCIES, EMERGENCY TRAINING, AND ERRORS

### 7.1 Emergencies

Despite sound clinical practice, emergencies, misadventures, and equipment faults will complicate and challenge routine clinical practice, often when least expected. It is important to understand the mechanisms and pathways of failure, have emergency procedures and practices in place for them, and rehearse and perform drills for optimal preparedness.

### 7.2 Types

An emergency occurs when an event or interruption takes place that interferes or disrupts an expected procedure. Emergencies may be anticipated or unanticipated but require action by the treatment team and a determination of whether the treatment can be completed as designed. A full accounting of potential error paths is beyond the scope of this report; however, general emergencies are discussed in three categories—environmental, patient‐related, and equipment.

The goal in providing a high‐level review of emergency types is to encourage sites to perform a robust review of different potential emergency scenarios and identify a plan for remediation and action for each.

1. Environmental

Environmental emergencies are challenges that derive from the facility and the overall environment of care. These include external natural events such as extreme weather like hurricanes, tornadoes, and tsunamis. During an incident, the facility infrastructure may be challenged, including loss of power, fires, internal flooding, or malfunctioning electromechanical systems such as treatment‐related equipment (e.g., vault doors, security systems, etc.). Staffing challenges may be presented by pandemics, strikes, rioting, or security events (e.g., active shooter) and can influence the team's ability to complete patient treatments. Excellent guidance is provided by the National Incident Management System (NIMS) used by the U.S. Federal Emergency Management Agency (FEMA) regarding incident management.^52^ The three important components of NIMS include resource management, command and coordination, and communication and information management. These components can be considered when developing emergency plans for BT patients. Policies and procedures must be developed to mitigate long‐term and short‐term risk and maintain patient safety. In many low‐lying areas, the threat of flood may require afterloaders to be moved out of basements and to a secure location on a higher floor. Patient care when sheltering in place should be thought through, such as needs for medications, generators, and medical equipment should other areas of the facility be inaccessible. Policies should be developed by a multidisciplinary team, and responsibilities clearly defined for incident command.

2. Patient Related

Patient‐related emergencies are events that result from a change in the condition of the patient. These events can manifest in different ways. On one extreme, a patient going through a BT procedure may experience a separate, traumatic, and urgent medical condition, for example, a myocardial infarction or a stroke. These events may require the treatment team to shift goals from radiation therapy to immediate life‐saving interventions. Alternatively, a confused or medicated patient may not be able to comply with instructions and could interfere with the treatment process, for example, attempt to remove an applicator or refuse to remain in position. For patients requiring anesthesia, medical and radiological emergency procedures should be reviewed. For physicists, the main responsibility is ensuring retraction of the HDR source and disconnection of the patient from the afterloader. The physicist may also help annotate the elapsed treatment time and interrupted treatment documentation.

3. Afterloader Specific

The potential for equipment faults is present during any use of the equipment. Minor equipment faults are faults that are expected to interrupt or delay treatment and may require staff or vendor interaction to correct. These faults include connection errors, source collision errors (e.g., from radius of curvature or bent/kinked catheters), or software glitches, and often can be overcome by corrections from the treatment team. Major equipment faults are more serious faults that require vendor interaction to clear. The key difference is being that a major fault will prevent treatment and often requires the replacement of system components. In an extreme case, a major afterloader fault may result in the source being stuck outside of the afterloader, requiring emergent removal and securing of the source‐applicator assembly and evacuation of the treatment room. Staff involved in HDR treatments should be familiar with common scenarios that would result in treatment interruption, such as machine interlocks, treatment error messages, and so on. A credentialing program for new staff is recommended and should include training for procedure‐specific interventions. At a minimum, refresher drills and emergency training should be conducted on an annual basis in accordance with 10 CFR 35.610(e).^51^

### 7.3 Emergency training

Training and competency of individuals were previously covered in section 6.2 of MMPG 13a Part A. Emergency training drills, as per NRC 35.610, should be performed initially during acceptance of the afterloader and annually by either the vendor (if written and agreed in the vendor contract), or by the licensee.^51^ Vendors can provide basic operational and safety training to users. In a “train‐the‐trainer” model, a vendor‐trained AMP, AU, or radiation safety officer (RSO) may subsequently provide training to other qualified individuals. Vendor‐provided training includes basic operational conditions, such as retracting an extended source, and basic responses in the event of equipment misadventure; however, an equally important aspect of emergency training is specific to the modalities and treatments in place at a given institution. While the vendor‐supplied emergency content is the basis for any facility's emergency procedures, a robust safety program should include applicator‐specific instructions in the event an applicator needs to be emergently removed from the patient and may, in some cases, deviate from the vendor's recommended steps. The highest standard of emergency training includes a drill during which an emergency is simulated, and the team will walk through (i.e., role‐play) an emergency response. Increasing the frequency of drills will reinforce critical concepts and maintain staff readiness, such that a review of roles and related actions could take place as frequently as every treatment session.

Per regulatory statute (see, e.g., Ref. ^51^), each licensed user must generate, implement, and maintain written procedures for responding to an emergency, and they must be posted in a visible and easily accessible area, for example, near the console.^51^ A flowchart or schematic may be included for concise, step‐by‐step response instructions. A complete procedure includes contact information and notification requirements for both the vendor (including site identification number, afterloader serial number, or equivalent) and for governing agencies, AUs, AMPs, and the RSO. The RSO should be notified of near‐misses and must be notified of all formal reports made to governing agencies to help assure compliance with regulations. A log of trained individuals must be retained in accordance with state or NRC regulations.

### 7.4 Emergency equipment

Emergency equipment must be readily available and depend on the types of procedures performed. Emergency equipment may be grouped into two categories: core emergency equipment useful in most situations and application‐specific equipment. Core emergency equipment items include: a portable survey meter, one or more long‐handled forceps, a storage container large enough to hold the largest applicator, a timer, and placards, signs, or warning indicators that may be deployed to secure the treatment room. Application‐specific/room‐specific equipment is strongly dependent on clinical practice, but examples include a syringe to deflate a breast BT balloon and/or surgical scissors or a suture removal kit to remove stitches.

Items such as flashlights, headlamps, masks, and gloves may be generally useful safety equipment for clinicians, especially in the event of a power loss or the need to expediently remove an applicator. While some afterloaders come with shielded storage containers or “emergency casks”, these containers may not be appropriately sized for all applicators or may be cumbersome in the event of an emergency. The largest shielded container available may be the HDR treatment room itself, which can be secured and appropriately posted once the patient and personnel have been evacuated from the area. A compromise may be found in the conversion of the secured HDR enclosure into a secured‐shielded enclosure that can serve in lieu of the cask in emergency scenarios and alleviate the need to remove the patient from the treatment room.

It is also imperative to confirm availability, proper functioning, and timely calibration of devices that will be used in the case of an emergency, for example, survey meter, indicators & alarms, personal dosimeter(s), flashlights, and stopwatches, among others. Annual emergency training may provide a good opportunity to perform this review, but it could also be incorporated into a new source acceptance or daily procedures.

### 7.5 Errors

A wide variety of papers describe error occurrence depending on the environment or procedure types that occur, with some procedures being more error‐prone than others.^53^, ^54^ In general, the rate of HDR BT errors is low (just 0.02% in 2009 and 2010), but they have the potential to be significant in terms of harm to the patient.^55^ Clinically used HDR sources usually range between 5 and 15 Ci (20,000 to 60,000 U). Consequently, the range of dose rates from such sources is approximately 25 to 75 cGy/s at 5 mm from the source. If the removal of the applicator from the patient is not done expediently, normal tissue tolerances can be exceeded. (see table V of AAPM TG59).^56^ It is therefore essential that staff can recognize and respond to radiation emergencies in a timely and efficient manner.

### 7.6 Error reporting

The prevention of errors and near misses is one of the most important tasks in a BT practice, however, there is always a risk that mistakes can happen. BT practitioners should adopt and use a patient safety reporting system.^57^ A variety of available error reporting and/or process improvement systems exist, such as RO‐ILS (Radiation Oncology Incident Learning System) from ASTRO, SAFRON (Safety in Radiation Oncology) from IAEA, RIRA (Radiotherapy Incident Reporting & Analysis System), or other in‐house systems. The goal of these systems is to provide a mechanism for shared learning from actual patient safety events and near‐misses. The use of error reporting systems has been shown to improve the quality of care by decreasing incidents and near misses.^58^ Not only should events that reach the patient be reported, but also near misses, which are extremely important for process improvement. A patient safety committee should review the submitted events, generate opportunities for team learning, and adopt specific advancements for patient safety.

The NRC and agreement states have specific requirements for reporting medical events, and those requirements must be followed. Similarly, radioactive materials licenses and commitments to regulatory agencies for specific reporting (e.g., as part of a corrective action plan) may also stipulate reporting that must be incorporated into practice.

### 7.7 Error analysis techniques

The report of AAPM TG‐100 describes the use of risk‐based analysis techniques to make radiation therapy delivery safer and more efficient.^59^ Briefly, the report describes the development of a quality management system, which includes the development of process maps, fault tree analysis, root‐cause analysis, identification of failure modes through Failure Mode and Effect Analysis (FMEA), assessment of risk, and implementing of interventions. BT practitioners could apply appropriate TG‐100 techniques to their clinical practice as a method to implement new safety mechanisms and improve patient safety. There are a multitude of publications regarding FMEA in HDR BT. Some focus on particular treatment sites while others focus on processes and equipment.^19^, ^47^, ^60^, ^61^, ^62^, ^63^, ^64^, ^65^ FMEA analysis may be informed by incident learning events as described by Paradis, et al.^66^ This methodology may be the best for long‐term process improvement.^67^ Many of these publications explicitly describe high‐risk processes. This MPPG provides safety counter‐steps to mollify those risks. The highest RPN (Risk Priority Numbers) were found collectively in the following steps:

- Incorrect digitization and reconstruction of needle/catheter pathways and/or tips
- Movement of needles or fixation devices during transport
- Wrong treatment channel lengths

MPPG 13a Part B has addressed the importance of checks to mitigate these errors as part of standard practice. As always, this guidance documents the *minimum* standard and encourages readers to develop processes and guidelines that are practical for the resources available.

## 8 CONCLUSIONS

This MPPG is the second part of MPPG 13A and describes the minimum practice guidelines for clinical applications of iridium‐192‐based HDR BT. General site‐specific guidelines tabulated the imaging and treatment practices and provided resources for further information. As always, users are encouraged to add additional tests and standards based on resources available.

## AUTHOR CONTRIBUTIONS

This guideline was reviewed and updated by the Medical Physics Practice Guideline Task Group 348 of the Professional Council of the AAPM. Each author reviewed recent literature on the topic and offered opinions on and language for the guideline. They also reviewed and applied comments from the full AAPM membership to the document.

This guideline was developed by the Medical Physics Practice Guideline Task Group 348 of the Professional Council of the AAPM. Each author reviewed recent literature on the topic and offered opinions on and language for the guideline. They also reviewed and applied comments from the full AAPM membership to the document. Each task group member has considerable experience in brachytherapy and provided their expertise to the recommendations herein.

## DISCLOSURE STATEMENT

The Chair of the report of TG‐348: Medical Physics Practice Guideline 13 on HDR Brachytherapy has reviewed the required Conflict of Interest statement on file for each author of the report of TG‐348 and determined that disclosure of potential Conflicts of Interest is an adequate management plan. Disclosures of potential Conflicts of Interest for each member of TG‐348 are found at the close of this report.

## CONFLICT OF INTEREST STATEMENT

The members of TG‐348 listed below attest that they have no potential Conflicts of Interest related to the subject matter or materials presented in this document.

Arjit Baghwala, MS

Gil'ad N. Cohen, MS

Claire Dempsey, PhD

Bruce Libby, PhD

Christopher S. Melhus, PhD

Robin A. Miller, MS

Samantha J. Simiele, PhD

The members of TG‐348 listed below disclose the following potential Conflict(s) of Interest related to subject matter or materials presented in this document.

Susan Richardson, PhD—Deputy Editor of the JACMP

Daniel J. Scanderbeg, PhD—consultant for Merit Medical Inc.
